# miR-526b-3p inhibits lung cancer cisplatin-resistance and metastasis by inhibiting STAT3-promoted PD-L1

**DOI:** 10.1038/s41419-021-04033-8

**Published:** 2021-07-28

**Authors:** Kuan-bing Chen, Wei Yang, Ying Xuan, Ai-jun Lin

**Affiliations:** 1 Department of Thoracic Surgery, Shengjing Hospital of China Medical University, Shenyang, China; 2 Department of Oncology, Shengjing Hospital of China Medical University, Shenyang, China; 3 Department of Radiology, Shengjing Hospital of China Medical University, Shenyang, China

**Keywords:** Cancer therapy, Translational research

## Abstract

Chemotherapy remains the primary treatment of advanced solid cancer, including lung cancer. However, as first-line treatment, cisplatin-based therapy is restricted by the frequent development of drug resistance. Increasing data showed that the programmed cell death protein ligand 1 (PD-L1) plays a vital role in regulating cisplatin resistance. However, the underlying mechanisms are not fully understood. We found that miR-526b-3p expression declined while PD-L1 was elevated in cisplatin-resistant lung cancer compared to that in cisplatin-sensitive lung cancer by analyzing clinical samples. Significantly, miR-526b-3p was associated with response to cisplatin negatively. We further demonstrated that miR-526b-3p reversed cisplatin resistance, suppressed metastasis, and activated CD8+ T cells in a STAT3/PD-L1-dependent manner. Thus, our findings extended the knowledge of PD-L1-mediated cisplatin resistance of lung cancer. In addition, the introduction of miR-526b-3p provided a new clue to improve the anti-tumor effects of the combination of immunotherapy and chemotherapy.

## Introduction

Platinum-based therapy is one of the most common chemotherapies against solid cancer, including lung cancer. Cisplatin, along with radiation therapy, is used to treat advanced lung cancer. Side effects and drug resistance are two main challenges that restrain the application and response of cisplatin [1]. Accumulating evidence shows that STAT3, MEK1, and AKT are frequently dysregulated in cisplatin-resistant cancer [2–4]. Therefore, therapies that targeted hyperactive genes are developed to diminish cisplatin resistance. However, the clinic outcomes of cisplatin-resistant cancer are not satisfactory.

miRNAs are endogenous noncoding RNA molecules that contain less than 25 nucleotides. miRNAs bind to the 3′-untranslated region (3′UTR) of target mRNAs, leading to mRNAs degradation and suppression. Therefore, miRNAs work as oncogenes and tumor suppressors in tumor malignancy. Nowadays, thousands of miRNAs molecules and corresponding target genes have been identified in the regulation of chemoresistance. For example, the interplays of long noncoding RNA HOXA11-AS1/miR-454/STAT3 drove chemoresistance of lung cancer [5]. miR-202 promoted cisplatin response in a RAS/MEK1 dependent manner [6]. Circulating miR-425 activated AKT and led to cisplatin resistance [7]. Due to one miRNA targets different mRNAs, while various miRNAs can regulate single mRNA, more knowledge is urgently needed to extend the knowledge of cisplatin resistance.

CTLA‐4, PD‐1, and PD‐L1 have been well studied and generated significant clinical benefits for advanced-stage cancer. PD-1 dominantly expresses in activated T cells and prevents T cells activation by interacting with PD-L1. PD-L1, the ligand of PD-1, expresses universally and increases in malignant cells [8]. Recent studies indicated that both PD-1 and PD-L1 contribute to cisplatin resistance. Kurimoto R and colleagues demonstrated that TGF-β/FGF2 promoted PD-L1 expression and conferred resistance against cisplatin [9]. Zhang P et al. found that PD-L1 reduction attenuated cisplatin resistance in lung cancer cells [10]. Accumulating data suggest that multiple signaling pathways merge in PD-L1 and confer lung cancer resistance against cisplatin afterward. Therefore, investigation of PD-L1-mediated cisplatin resistance is expected to provide a novel rationale for treating refractory lung cancer.

In the present study, we aimed to figure out the association of miR-526b-3p, STAT3, and PD-L1 in cisplatin-resistant lung cancer by collecting clinical information. We further attempted to clarify the details of miR-526b-3p/STAT3/PD-L1 signaling pathways-mediated resistance by conducting gain-of-function and loss-of-function experiments.

## Material and methods

### Patients’ recruitment and tissue samples collection

One hundred non-small-cell lung cancer patients who had received chemotherapy were recruited into the present study. Those patients who were diagnosed with synchronous distant metastasis, received targeted therapy, or immune checkpoint inhibitors were excluded. The cancer tissues were reviewed by two independent pathologists, and the complete pathological information was collected (Table 1). Written consent for research and publication was obtained from each participant. The study was approved by the Medical Ethics Committee of Shengjing Hospital of China Medical University.

**Table 1:** The correlation between miR-526b-3p expression and pathological features of lung cancer.

Parameters	Description	No. of patient	miR-526 expression		χ^2^	*P* value
			Low	High		
Gender	Male	67	45	11	3.243	0.0717
	Female	33	16	17		
Age (years)	<50	39	21	18	1.375	0.2409
	≥50	61	40	21		
Lymph node metastasis (pN)	No	8	2	6	4.737	0.0295
	Yes	92	59	33		
TNM stage	I	8	2	6	8.16	0.0169
	II	23	11	12		
	III	69	48	21		
Response to cisplatin	Resistant	50	39	11	12.15	0.0005
	Sensitive	50	22	28		

*P* = 0.05 was considered statistically significant.

### miRNA microarray analysis

RNA gained from tumor tissues was used for custom miProfile^TM^ Cancer miRNA qPCR Array (Cat. No. QM001-E, GeneCopoeia, Rockville, MD, USA). The methods for gathering RNA and synthesizing cDNA were described in the qRT-PCR section. qPCR arrays were conducted following the vendor’s protocol.

### Cell lines and reagents

Human epithelial virus-transformed lung bronchus cell line BEAS-2B (ATCC^@^CRL-9609^TM^), human lung carcinoma cell LINE H1975 (ATCC^@^CRL-5908TM), A549 (ATCC^@^CRL-185^TM^) were provided by American Type Culture Collection (Manassas, VA, USA). PC-9 (formerly known as PC-14, Code: 90071810) was supplied by the European Collection of Cell Cultures (ECACC; Salisbury, United Kingdom). Cisplatin-resistant A549 (A549/DDP) and cisplatin-PC-9 (PC-9/DDP) were gifts from Dr. Xuejun Guo. BEAS-2B cells were grown in RPMI-1640 with 10% fetal bovine serum. The rest cells were cultured in Dulbecco’s modified Eagle medium (DMEM) containing 10% fetal bovine serum. Cisplatin-resistant cells were maintained with DMEM plus 0.1 μg/ml cisplatin as previously described [11]. Medium and serum were obtained from Sigma-Aldrich (St. Louis, MO, USA). Mycoplasma Detection Kit (Cat.No.MP0025, Sigma-Aldrich) was used to detect Mycoplasma during cell culture.

Cisplatin (Cat.No.C2210000) was purchased from Sigma-Aldrich. miR-526b-3p, miR-526b-3p inhibitor, and empty vector pEZX-MR04 were purchased from GeneCopoeia. pDONR223_STAT3_WT was a gift from Jesse Boehm & William Hahn & David Root (Addgene plasmid # 82235; http://n2t.net/addgene:82235; RRID: Addgene_82235) [12]. Vector pDONR223 was generated by Invitrogen (Cat.No.12536017, Carlsbad, CA, USA).

### Putative miRNA and mRNA interactions

The candidate sponge mRNAs for miRNAs were predicted by miRDB online database (http://www.mirbase.org/) [13, 14].

### Cell growth assay

MTT (3-[4,5- dimethylthiazol-2-yl]-2,5-diphenyl tetrazolium bromide) based-colorimetric assays were carried out adapted to the manufacturer’s protocol (Sigma-Aldrich, St. Louis, MO, USA). A total of 0.8 × 10^4^ cells were plated in 96-well plates with 0.1 ml DMEM. Cells were treated with cisplatin at different doses. Finally, cells were incubated with ten μl MTT solution (5 mg/ml) for four hours at 37 ˚C prior to analysis. The optical densities were read at 490 nm using a Microplate Reader (Thermo Fisher Scientific, Waltham, MA, USA).

### Migration assay

Transwell migration assay was performed using modified Boyden chambers (Sigma-Aldrich). A total of 4 × 10^4^ cells in serum-free medium were plated in the upper room of each chamber, whereas the lower room was filled with medium supplemented with 10% fetal bovine serum. After incubating for 18 h, cells in the upper compartments were discarded. In contrast, the migrated cells in the lower parts were stained with 1% crystal violet solution, followed by counting under Olympus microscope CX23 (Shinjuku, Tokyo, Japan).

### Western blots

Protein was collected by whole-cell lysis buffer. Forty micrograms protein was resolved with SDS-PAGE gel and was transferred to Polyvinylidene fluoride membranes (Sigma-Aldrich). The membranes were blocked with 5% skim milk for 1 h and incubated with the following primary antibodies overnight at a dilution of 1:1000. STAT3 (Cat.No.9139), PD-L1 (Cat.No.13684), c-Myc (Cat.No.5605), MDR1 (Cat.No.13342), and GAPDH (Cat.No.5174) were purchased from Cell Signaling Technology (Danvers, MA, USA). The membranes were washed with TBST and incubated with HRP-conjugated secondary antibodies (Cell Signaling Technology) at a dilution of 1:2000 for 1 h. Protein was visualized using the Western Lighting Ultra (Thermo Fisher Scientific).

### Real-time quantitative polymerase chain reaction (RT-qPCR)

RNA was extracted by RNA isolation kit (Cat.No.83913, Sigma-Aldrich) per the manufacturer’s protocol. Two hundred fifty nanograms amount of RNA was reverse transcribed to cDNA by a Reverse Transcription Reaction kit (Qiagen, Hilden, Germany). RT-qPCR was carried out using the LightCycler^®^480 System (Roche, Basel, Switzerland). PCR was conducted as follows: 40 cycles of 94 °C for 18 s, 60 °C for 15 s, and 72 °C for 30 s. All procedures were repeated independently. Gene expression was normalized to *GAPDH* using the 2^−ΔΔCq^ method [15]. All primers were synthesized by Genechem (Shanghai, China). The primer sequences for detecting the indicated genes were listed below.

*miR-526b-3p*, F,5′-CTCTTGAGGGAAGCACT-3′; R, 5′-GAACATGTCTGCGTATCTC-3′. *STAT3*, F,5′-CTTTGAGACCGAGGTGTATCACC-3′; R, 5′-GGTCAGCATGTTGTACCACAGG-3′. *PD-L1*, forward,5′-TGCCGACTACAAGCGAATTACTG-3′; R, 5′-CTGCTTGTCCAGATGACTTCGG-3′. *MDR1*, forward,5′-GCTGTCAAGGAAGCCAATGCCT-3′; R, 5′-TGCAATGGCGATCCTCTGCTTC-3′. *c-Myc*, forward,5′-CCTGGTGCTCCATGAGGAGAC-3′; R, 5′-CAGACTCTGACCTTTTGCCAGG-3′. *GAPDH*, F,5′-GTCTCCTCTGACTTCAACAGCG-3′; R, 5′-ACCACCCTGTTGCTGTAGCCAA-3′.

### Luciferase reporter assay

1-1020 of 3′untranslation region of STAT3 was inserted into pDONR223. miR-526b-3p or miR-526b-3p mutant was inserted into pmirGLO (Cat.No. E1330, Promega, Madison, WI, USA) according to the vendor’s instructions. Twenty-four hours before transfection, cells were plated in 96-well dish plates at a density of 2 × 10^4^ per well. pmirGLO-miR-526b-3p, pmirGLO-miR-526b-3p mutant (miR-526b-3pMUT) along with pDONR223_STAT3 3’UTR were introduced into cells prior to dual-luciferase reporter assay. Luciferase activity was determined post-24 h transfection with the Luc-Pair™ Duo-Luciferase Assay Kit 2.0 (GeneCopoeia).

### Immunohistochemical staining

The STAT3 primary antibody (Cat.No.9139) was used to detect STAT3 expression in formalin-fixed, paraffin-embedded tissues according to the previous protocol [16]. Briefly, antigen was retrieved in citrate buffer (10 mM, pH 6.0), washed with phosphate-buffered saline, and exposed to 3% hydrogen peroxide. Slides were incubated with anti-STAT3 at a dilution of 1:200 at room temperature for one hour. Elivision^TM^ plus Polymer HRP (Mouse/Rabbit) IHC Kit (Cat.No.9901, Maxin, Fuzhou, China) was used to visualize STAT3 stains adapted to the vendor’s instruction.

### A549 metastatic xenograft models

Five-week-old BALB/c male nude mice were purchased from Beijing Laboratory Animal Research Center (Beijing, China). Mice were housed in conditions under the guidelines of the experimental animal center of Shengjing Hospital. A total of 5 × 10^5^ A549 cells were intravenously injected into fifteen nude mice. Mice were separated into three groups randomly (five per group) seven days later. Twenty-eight days post-injection, mice were sacrificed with carbon dioxide inhalation. Hematoxylin and eosin staining was conducted according to the standard procedure afterward [17]. Additionally, gene expression was accessed by Western blots and qPCR assay. All studies were approved by the medical ethics committee of Shengjing hospital of China Medical University. We complied with the guideline of the Experimental Animal Center of Shengjing Hospital of China Medical University during the generation of animal models.

### Statistical analysis

Data in graphs were shown as mean ± standard deviation (SD). The association of miR-526b-3p and STAT3 was analyzed by Spearman’s correlation coefficient test. The differences among groups were evaluated by one-way or two-way Analysis of Variance, following by a Sidak’s multiple comparisons test. *P* < 0.05 indicated statistical significance. Statistical analysis was performed by using GraphPad version 8.1 (San Diego, CA, USA).

## Results

### miR-526b-3p decreases in cisplatin-resistant lung cancer

**Fig. 1:**
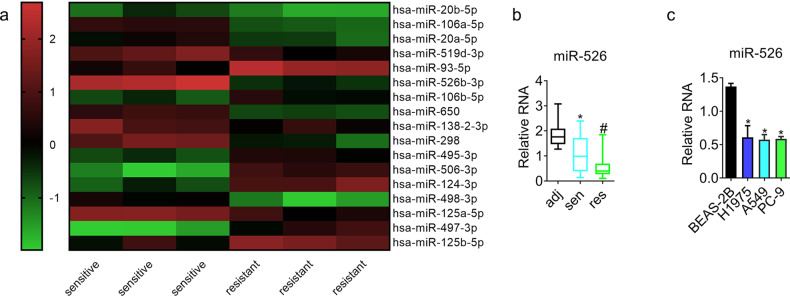
miR-526b-3p declines in cisplatin-resistant lung cancer. **a** The differential expression of miRNAs between cisplatin-sensitive and cisplatin-resistant lung cancer is analyzed by miRNA microarray. The color indicates the fold change of the target gene normalized to the corresponding housekeeping genes. **b**, **c** miR-526b-3p expression in tumor specimens and cell lines is determined by qRT-PCR. adj adjacent tissues; sen cisplatin-sensitive tumor tissues; res cisplatin-resistance tumor tissues. *, *P* < 0.05 vs. adjacent tissues or BEAS-2B cells. #, *P* < 0.05 vs. cisplatin-sensitive tissues.

To explore the differential expression of miRNAs in cisplatin-resistant lung cancer, we conducted miRNA arrays with three cisplatin-resistant and three cisplatin-sensitive specimens. Fig 1a exhibited that miR-526b-3p dropped in cisplatin-resistant tissues. We further demonstrated that miR-526b-3p fell significantly in 50 cisplatin-resistant cases compared to that in cisplatin-sensitive cases (Fig. 1b). The results in Fig. 1c showed that miR-526b-3p declined in lung cancer cell lines compared to the immortalized human non-tumorigenic lung epithelial cell line BEAS-2B. We selected A549 and PC-9 as cell models in the following in-vitro experiments due to there was no significant difference in miR-526b-3p expression.

### miR-526b-3p suppresses lung cancer malignancy

**Fig. 2:**
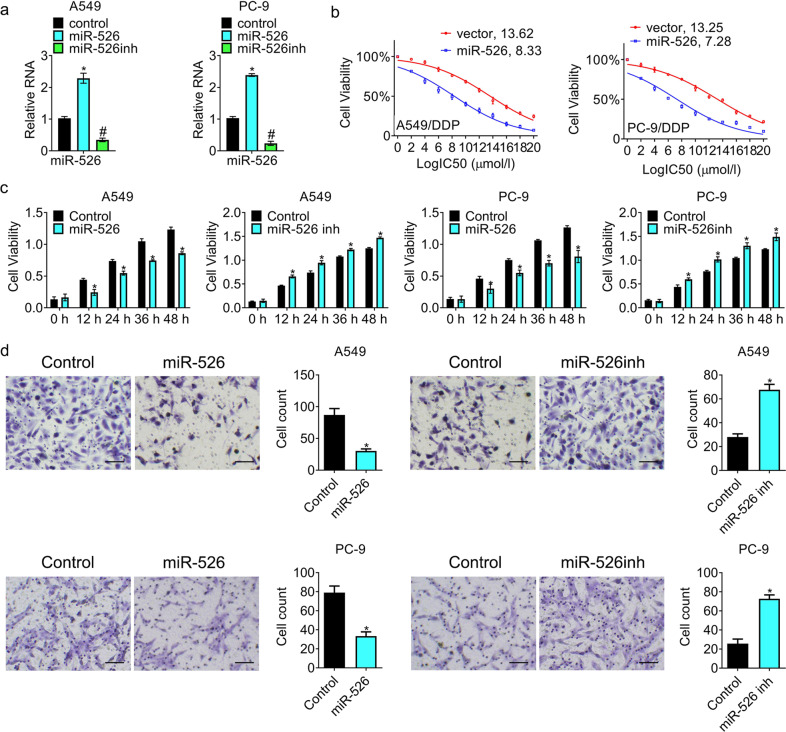
miR-526b-3p overexpression suppresses resistance against cisplatin, cell growth, and migration of lung cancer. **a** The expression of miR-526b-3pin cells is accessed by qRT-PCR. **b** The viability of cells with miR-526b-3p post cisplatin exposure is determined by MTT assay. **c** The growth of the indicated cells is detected by MTT assay. **d** The migration of the indicated cells is determined by a transwell assay. *, *P* < 0.05 vs. control.

To investigate the effects of miR-526b-3p in lung cancer cells, we introduced miR-526b-3p mimic (miR-526) and miR-526b-3p inhibitor (miR-526inh) into A549 and PC-9 cells. The results in Fig. 2a exhibited that expression of miR-526b-3p increased in cells post-transfection of miR-526b-3p mimic, while that decreased in cells with miR-526b-3p inhibitor. Besides, we introduced miR-526b-3p mimic into cisplatin-resistant cells, following by exposure to cisplatin. Fig 2b demonstrated that the IC_50_ values against cisplatin in the miR-526b-3p group were reduced compared to the vector. Besides, ectopic expression of miR-526b-3p suppressed cell viability while miR-526b-3p inhibitor promoted cell viability (Fig. 2c). Moreover, miR-526b-3p overexpression repressed cell migration, whereas miR-526b-3p knockdown enhanced cell migration (Fig. 2d). The results demonstrated that miR-526b-3p was a tumor suppressor in cisplatin-resistant lung cancer.

### STAT3 is one of the targets of miR-526b-3p and increases in cisplatin-resistant lung cancer

**Fig. 3:**
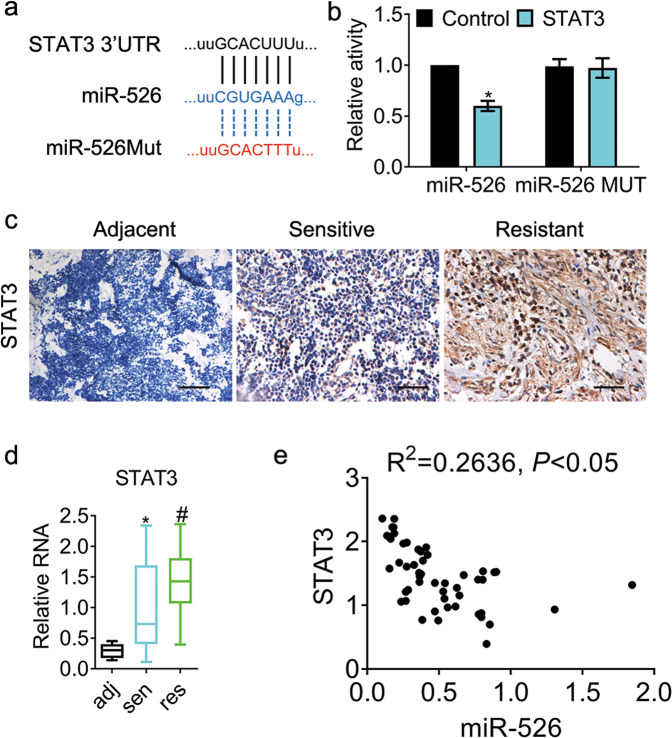
STAT3 is a target of miR-526b-3p and increases in cisplatin-resistant lung cancer. **a** The putative binding sites of STAT3 3′UTR and miR-526b-3p are shown. **b** The relative dual-luciferase activity of STAT3 3′UTR in the indicated cells is measured. **c** The representative images of STAT3 expression in lung cancer tissues are shown. Sensitive, cisplatin sensitive. Resistant, cisplatin-resistant. Scale bar, 50 µm. **d** The expression of STAT3 in lung cancer tissues is accessed by qRT-PCR. *, *P* < 0.05 vs. adjacent tissues. #, *P* < 0.05 vs. cisplatin-sensitive tissues. **e** The correlation of STAT3 and miR-526b-3p is estimated by Spearman’s correlation coefficient. *, *P* < 0.05.

We used miRDB database to predict potential candidates of miR-526b-3p. Signal Transducer and Activator of Transcription 3 (STAT3) exhibited the most upregulation among hundreds of candidate genes (Supplementary Figure). We, therefore, selected STAT3 to investigate further. Fig 3a showed the schematic diagram of the binding sites between miR-526b-3p and STAT3 3′UTR. We generated the miR-526b-3p (miR-526b-3pmut) mutant, which was insufficient to adhere to the 3′UTR of STAT3. The 3′UTR of STAT3 plus miR-526b-3p, or miR-526b-3pmut, were introduced into cells, followed by dual-luciferase assays. The results in Fig. 3b showed that the 3′UTR of STAT3 changed little in the miR-526b-3pmut group, while that in the miR-526b-3p group declined compared to control. In addition, we detected the expression of STAT3 in lung cancer. Fig 3c exhibited that the STAT3 expression in cisplatin-resistant lung cancer was higher than that in cisplatin-sensitive specimens. We also examined STAT3 expression in cancer tissues by qRT-PCR. The results were in line with those of immunohistochemical staining (Fig. 3d). Furthermore, STAT3 expression was negatively correlated with miR-526b-3p (Fig. 3e). The results indicated that miR-526b-3p inhibited STAT3 in cisplatin-resistant lung cancer.

### miR-526b-3p/STAT3 axis regulated lung cancer malignancy

**Fig. 4:**
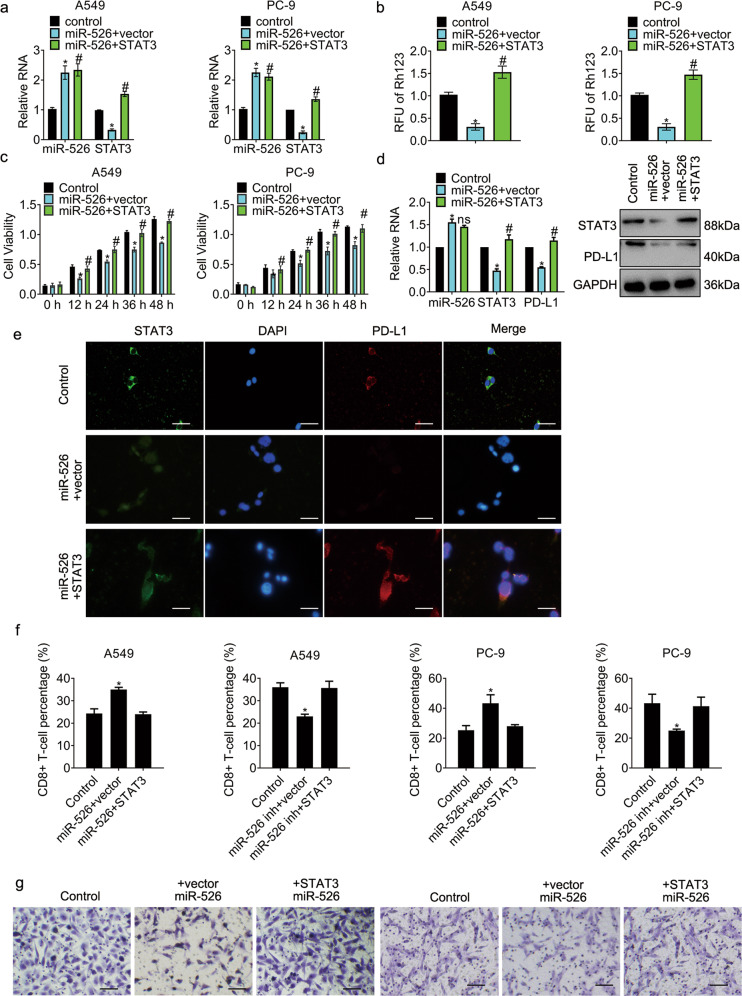
STAT3 overexpression overwhelms the inhibition of miR-526b-3p in cancer cells. **a** miR-526b-3p and STAT3 expression is accessed by qRT-PCR. **b** The activity of MDR1 is measured by rhodamine123 efflux assay. RLU relative fluorescence of unit. **c** The viability of the indicated cells is accessed by MTT assay. **d** The expression of the indicated genes is detected by qRT-PCR and Western blot, separately. **e** Representative immunofluorescent images of STAT3 and PD-L1 in A549 cells are shown. **f** The percentage of CD8 positive T-cells in the indicated cells are accessed by flow cytometry analysis. **g** The motility of the indicated cells was detected by transwell migration assay. Scale bar, 100 µm. *, *P* < 0.05 vs control. #, *P* < 0.05 vs miR-526b-3p plus vector.

To explore the role of the miR-526b-3p/STAT3 axis in the regulation of lung cancer malignancy, we introduced miR-526b-3p and STAT3 into cells. Fig 4a showed that STAT3 expression fell in cells post miR-526b-3p transfection, whereas that augmented in cells with the combination. The relative fluorescent unit of rhodamine123 in the miR-526b-3p group was reduced, whereas that in the combo escalated compared to control (Fig. 4b), suggesting that the ectopic expression of STAT3 reversed the inhibition of miR-526b-3p in drug efflux. The viability of cells with miR-526b-3p declined, while that of cells with the combo increased (Fig. 4c). The results proved that STAT3overexpression attenuated the suppression of miR-526b-3p in cell growth. Besides, miR-526b-3p inhibited the expression of programmed death-ligand 1 (PD-L1), while the combo promoted PD-L1 expression (Fig. 4d, e). Because PD-1/PD-L1 interaction enhances the immune evasion in tumors by inhibiting CD8+ T cells [18], we wondered whether the population of CD8+ T cells was interfered with by the introduction of miR-526b-3p. The results in Fig. 4f exhibited that miR-526b-3p triggered the population of CD8+ T cells, whereas STAT3 interrupted the promotion. The motility of cells in the miR-526b-3p group was reduced while that in the combo amplified compared to control (Fig. 4g).

### miR-526b-3p/STAT3 axis regulated cisplatin resistance in a PD-L1-dependent manner

**Fig. 5:**
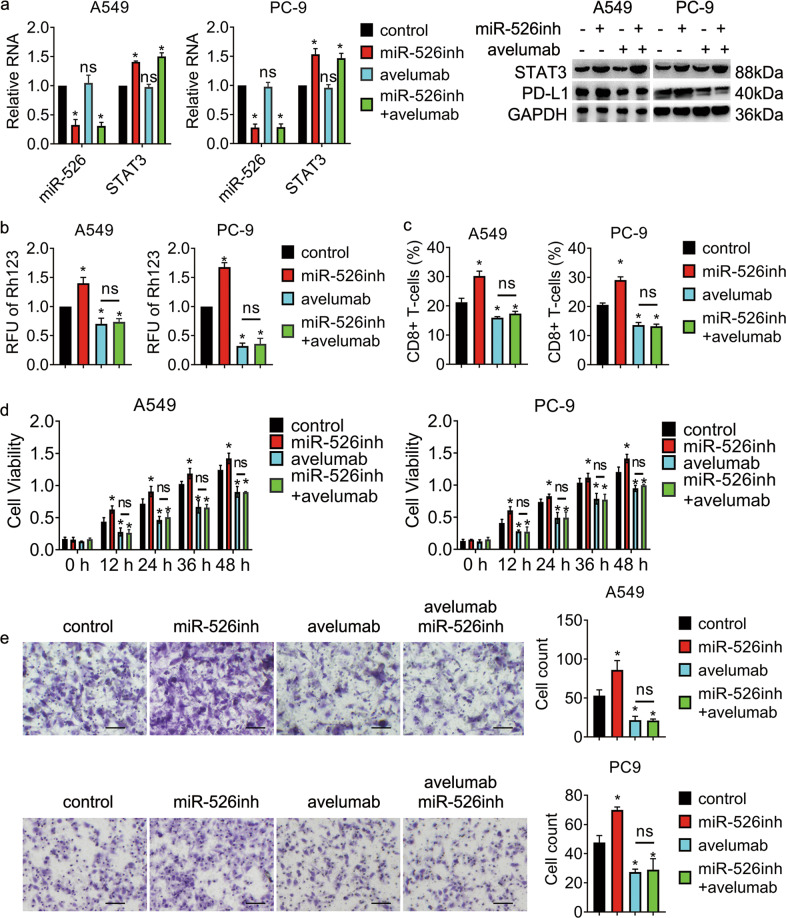
miR-526b-3p/STAT3 axis regulates cisplatin resistance in a PD-L1-dependent manner. **a** Expression of miR-526b-3p, STAT3, and PD-L1 is accessed by qRT-PCR and western blot, separately. **b** The activity of MDR1 is measured by rhodamine123 efflux assay. RLU relative fluorescence of unit. **c** The percentage of CD8 positive T-cells in the indicated cells are accessed by flow cytometry analysis. **d** The viability of the indicated cells is accessed by MTT assay. **e** The motility of the indicated cells is detected by transwell migration assay. Scale bar, 100 µm. *, *P* < 0.05 vs. control. ns no significance vs avelumab.

Previous studies have shown that STAT3 promotes PD-L1 expression, contributing to chemoresistance of breast cancer, head, and neck squamous cell carcinoma, and non-small-cell lung cancer [19–21]. We, therefore, investigated the effects of miR-526b-3p and STAT3 in PD-L1 expression. The results in Fig. 5a showed that the knockdown of miR-526b-3p promoted STAT3 and PD-L1 expression. Avelumab, a monoclonal antibody that targets PD-L1, significantly suppressed PD-L1 while having little effect in STAT3 or miR-526b-3p expression. The relative fluorescent unit of rhodamine123 in the miR-526b-3p inhibitor group increased, whereas that in the avelumab group dropped compared to control. Notably, avelumab repressed the effects of miR-526b-3p inhibitor in drug efflux (Fig. 5b). In addition, the population of CD8+ T cells in the miR-526b-3p inhibitor group fell, whereas that in the combination was similar to the avelumab group (Fig. 5c). Further, the viability of miR-526b-3p-knockdown cells developed while that of the combination group remained the same as the avelumab group (Fig. 5d). Moreover, miR-526b-3p inhibitor enhanced cell migration while avelumab repressed cell motility. Importantly, the cell migration of the combo group was identical to that of avelumab (Fig. 5e). The results indicated that avelumab treatment abolished miR-526b-3p-mediated anti-tumor effects. Thus, PD-L1 was a new downstream target of miR-526b-3p/STAT3 mediated cisplatin of lung cancer.

### miR-526b-3p/STAT3 regulated cancer metastasis in vivo

**Fig. 6:**
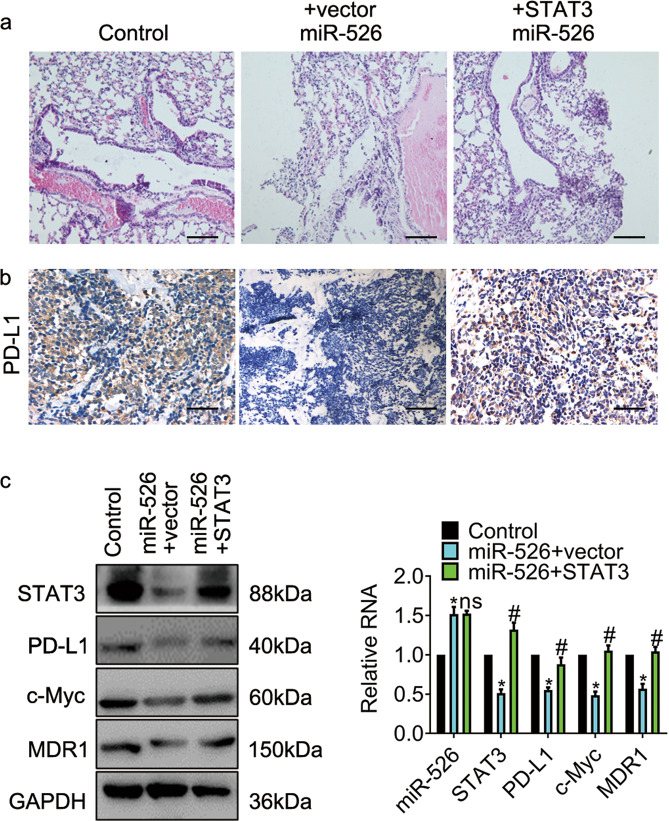
STAT3 overexpression reverses the inhibition of miR-526b-3p in cancer metastasis. **a** The representative images of lung metastasis by hematoxylin and eosin stains are shown. Scale bar, 20 µm. **b** The representative images of PD-L1 expression by immunohistochemical stain are shown. Scale bar, 50 µm. **c** The expression of the indicated genes is determined by western blot and qRT-PCR, respectively. *, *P* < 0.05 vs. control. #, *P* < 0.05 vs miR-526b-3p plus vector.

We generated A549 mouse metastatic models with cells carrying control, miR-526b-3p, miR-526b-3p plus STAT3. Lung metastasis was validated by hematoxylin-eosin stains (Fig. 6a). The results in Fig. 6b demonstrated that PD-L1 expression declined in the miR-526b-3p group, while that increased in the combination group. Besides, we found the oncogene c-Myc and the multidrug resistance regulator MDR1 decreased in the miR-526b-3p group, whereas those increased in the combo group (Fig. 6c). Thus, the observations obtained in the animal models were in line with the results gained in vitro.

## Discussion

Chemotherapy remains the preferred and primary treatment for advanced lung cancer. Cisplatin is widely used as the first-line medicine. Unfortunately, the frequent development of resistance prevents the continuous use of cisplatin and leads to treatment failure. Multiple mechanisms, such as gene mutation, abnormal gene expression, and miRNA dysregulation, play vital roles in developing cisplatin resistance. Increasing data have shown that miRNA expression in cisplatin-resistant cancer cells and the corresponding parental cancer cells are distinct. We found that miR-526b-3p overexpression enhanced the response to cisplatin while attenuating cell growth and migration in cisplatin-resistant lung cancer cells. The observations that miR-526b-3p as a tumor suppressor were supported by previous studies. For example, Ming W et al. demonstrated that miR-526b-3p targeted WEE1, leading to glioma regression [22]. Furthermore, Zhang R et al. proved that miR-526b-3p retarded colon cancer metastasis by suppressing HIF1α [23]. Besides, the ectopic expression of miR-526b-3p interrupted malignancies by forming complexes with long noncoding RNAs and mRNAs [24–26].

The aberrant activation of STAT3 is correlated to poor clinical outcomes of lung cancer [27]. The dysregulation of fibroblast growth factor receptor, vascular endothelial growth factor, IL-6, and extracellular signal-regulated kinase contribute to hyperactivation of STAT3. Particularly, STAT3 is one of the pivotal factors that cause cisplatin resistance. Tremendous studies have indicated that STAT3 is a promising therapeutic target for conquering cisplatin resistance [28, 29]. Recently, various miRNAs are emerging as critical regulators of STAT3. For example, miR-608 attenuated lung cancer malignancy in a JANK2/STAT3 dependent manner [30]. miR-146b-3p promoted cervical cancer cell proliferation and migration via STAT3 and AKT signaling pathways [31]. We demonstrated that miR-526b-3p directly targeted STAT3 and subsequently inhibited the expression of PD-L1, c-Myc, and MDR1. Apart from miR-526b-3p, STAT3 expression is regulated by different miRNA. Previous research has shown that miR-296-5p, miR-106-5p, miR-10b, and miR-21 target STAT3 by adhering to STAT3 3’UTR, contributing to cisplatin resistance [32–35]. The reasons for the divergence of miRNA-STAT3 interactions are not fully understood yet. The alternative genome in various cancer can be one of the explanations.

Previously, Shen M et al. proved that Ataxia Telangiectasia Mutated (ATM) promoted PD-L1 expression by enhancing JAK/STAT3 signaling cascades in lung cancer [36]. Zhang P et al. revealed that the IL-6/STAT3 axis conferred head and neck squamous cell carcinoma resistance against cisplatin [37]. Furthermore, Lei Z et al. found that the combination of anti-PD-L1 Atezolizumab and anti-vascular endothelial growth factor Bevacizumab prevented cisplatin-resistant ovarian cancer progression by suppressing STAT3-induced epithelial-mesenchymal transition [38]. The results mentioned above were consistent with our present results, indicating that STAT3/PD-L1-mediated cisplatin resistance is universal. PD-L1 elevates in various cancer cells post-chemotherapy and subsequently promotes cancer progression by facilitating immune evasion and chemoresistance [39]. A previous study showed that let-7-inhibited PD-L1 expression reactivated CD8+ T cells and reversed cisplatin resistance of non-small-cell lung cancer [40]. We demonstrated that the avelumab treatment abrogated the effects of miR-526b-3p inhibitor, providing new clues to draw a global picture of PD-L1-mediated chemoresistance.

Briefly, miR-526b-3p targeted STAT3 and inhibited cell growth, migration, and cisplatin resistance in a PD-L1-dependent manner. miR-526b-3p may be a potential therapeutic target for improving the anti-tumor effects of chemo-immunotherapy combination.

## Supplementary information

Figure supplementary

## Data Availability

The RT-PCR profiling analysis of the arrays is curated by Gene Expression Omnibus (https://www.ncbi.nlm.nih.gov/geo/query/acc.cgi?acc=GSE168707).
