# Supplementary figures and images for: miR-526b-3p inhibits lung cancer cisplatin-resistance and metastasis by inhibiting STAT3-promoted PD-L1
Source: Cell Death Dis. 2021 Jul 28;12(8):748. doi: 10.1038/s41419-021-04033-8 (PMC8319181; doi:10.1038/s41419-021-04033-8)

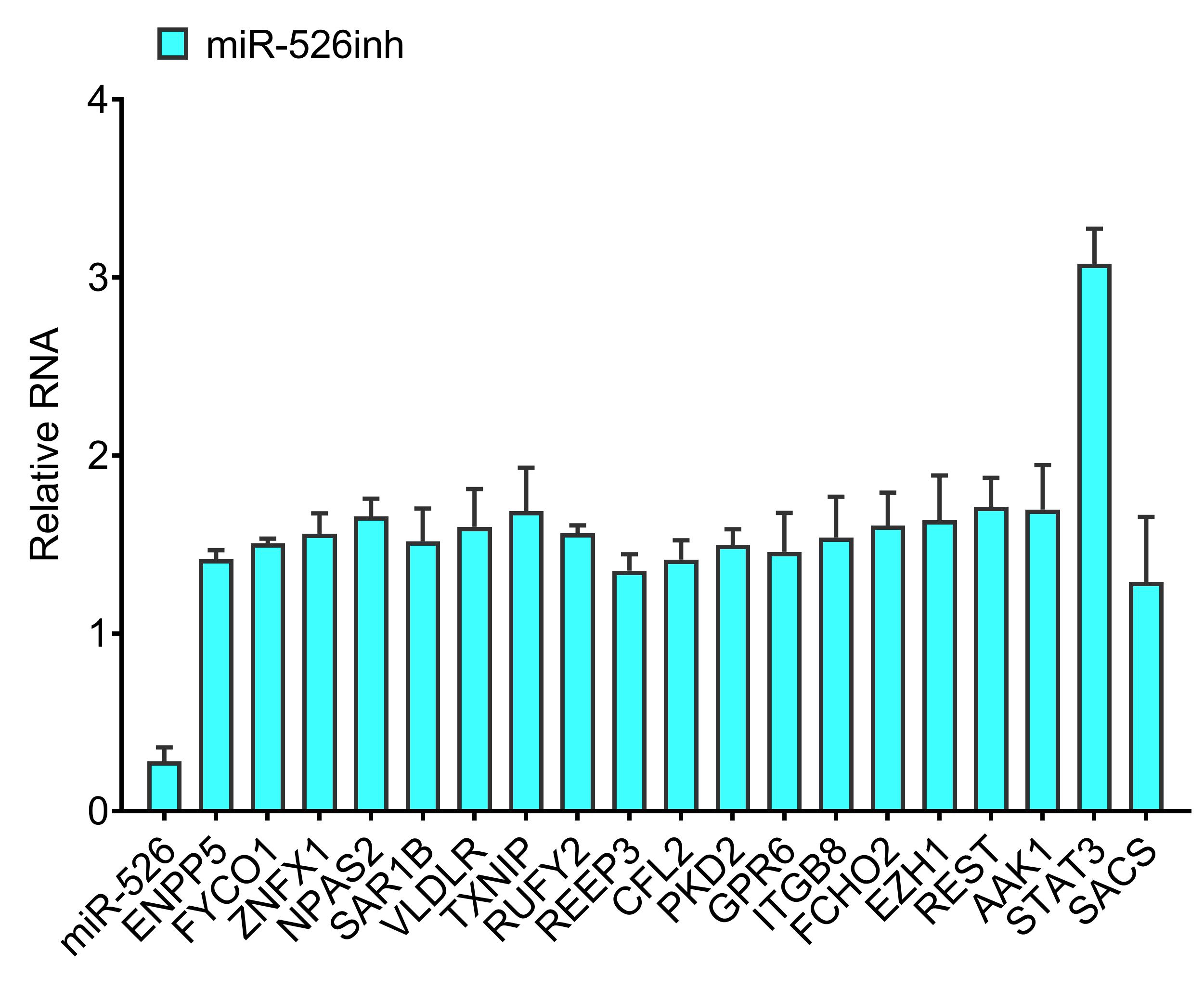

Supplement: Supplementary file 1 — Figure supplementary [file 41419_2021_4033_MOESM1_ESM.jpg]
